# Surgical replacement of iatrogenically prolapsed penis in a dromedary camel

**Published:** 2012-11-28

**Authors:** M.I. Siddiqui, M.N. Telfah, S.A.T. Al-Qubati

**Affiliations:** *Central Veterinary Hospital, Al-Wathba, Abu Dhabi, United Arab Emirates*

**Keywords:** Paraphimosis, Penile prolapse, Preputial cavity, Preputial orifice

## Abstract

Prolapse of the penis through an iatrogenic incision on the right side of the preputial base in a five year old dromedary camel was handled surgically and the organ was successfully replaced into the preputial cavity. The condition occurred as a result of draining an abscess at the base of the prepuce by a quack about eight months earlier. The reason to report this case lies in its peculiarity that although the penis remained outside the preputial cavity for about eight months exposed to the external environment, yet no complications pertaining to its fragile tissue and urination occurred during this long period as seen in cases of paraphimosis.

## Introduction

The prolapse of penis through the preputial orifice (paraphimosis) has been reported in the camel where the organ usually undergoes strangulations, necessitating partial penectomy in the perineal region (Choudhary *et al.*, 1981). In such cases, the replacement of the penis in the preputial cavity is difficult due to the size, shape and direction of the prepuce and prescrotal position of the sigmoid flexure in the camel (Smuts and Bezuidenhout, 1987; Gahlot, 1996) as compared to that in the bovine bull (Walker, 1979). This difference should be kept in mind while carrying out surgical manipulations on this organ.

This report describes successful replacement of the prolapsed penis into the preputial cavity that occurred through an iatrogenic opening as a result of earlier abscess drainage by a quack at the base of the prepuce.

## Case Details

**Fig. 1:**
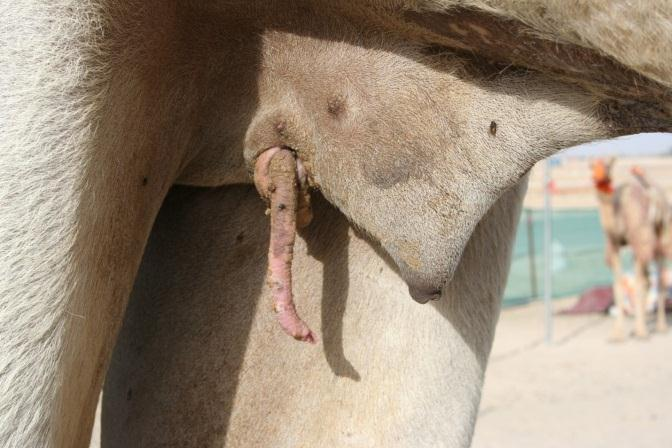
Prolapse of penis through the base of the prepuce.

A five year old dromedary camel with prolapse of the penis through an iatrogenic opening at the right side of the base of the prepuce was examined at the Al-Wathba race area in the emirate of Abu Dhabi (Fig. 1).

The history revealed that an abscess at this site was drained by a quack at a far off place in the desert of Abu Dhabi and the penis prolapsed through the incision two days after. Apparently, the incision was quite deep up to the preputial cavity that allowed the prolapse to occur.

The present owner got this animal as a gift and contacted the hospital for necessary corrective surgery. At the time of presentation, the case - according to the owner - was about 8 months old. The animal had normal food and water intake with normal defecation and urination and was quite healthy with a normal blood count (Table 1).

**Table 1:** Hematology report of the camel.

Test	Results	Ref. Range	
HCT	29	25-35	%
HGB	13.0	10.2-16.0	g/dl
RBCs	10.3	7.5-12.0	10^6/ul
MCV	27.7	27.0-33.0	fl
MCH	12.6	12.5-16.5	pq
MCHC	45.5	42.0-49.6	q/dl
WBCs	12.2	7.0-16.0	10^3/ul
NEUT	51.9	30.0-60.0	%
LYMPH	37.5	30.0-55.0	%
MONO	5.5	2.0-6.0	%
EOSINO	4.4	2.0-8.0	%
BASO	0.7	Up to 2.0	%

Hematology report generated by the Laboratories Unit, Central Veterinary Hospital, AL Wathba, Abu Dhabi, United Arab Emirates.

There were no signs of inflammation, edematous swelling or any tissue damage to the organ except a walnut size granulomatous mass on the posterior edge of the defect.

Although the penis was slightly smeared with sand particles, its tissue retained the normal texture. Under intravenous anesthesia of 2% xylazine hydrochloride (Xyla-Ject, ADWIA Co. S.A.E. Egypt) and 10% ketamine hydrochloride (Ketamine 10%, Alfasan, Woerden, Holland) at a dose rate of 0.4 mg/kg of body weight of each drug, a blunt pointed long forceps introduced into the preputial cavity through the preputial orifice stuck at the defect indicating complete healing of its anterior margin thus blocking the way to the preputial cavity.

A 360 degree digital manipulation of the defect around the penis revealed a sort of fibrous ring that did not allow further examination. This together with the presence of the granulomatous mass was an indication that the wound was quite old that had undergone healing around the penile body without exerting any undue pressure on the organ.

### Control and Anesthesia

The animal was kept fasting for 24 hours prior to surgery. For the purpose of precising the dose of anesthesia, weight of the animal was calculated approximately 300 kg, using the formula described by Kohler-Rollefson *et al*. (2001), where:

Live body weight (Kg) = shoulder height (m) x chest girth (m) x hump girth (m) x 50 Kg.

The animal was controlled in the sternal recumbency and just before surgery, was deeply sedated with a mixture of 2% solution of xylazine hydrochloride (Xyla-Ject, ADWIA Co. S.A.E. Egypt) and 10% solution of ketamine hydrochloride (Ketamine 10%, Alfasan, Woerden, Holland) given intravenously at the dose rate of 0.4 mg/kg of body weight – of each drug (Abrahamsen, 2009; Siddiqui and Telfah, 2010). The calculated dose came to be 6 ml of xylazine hydrochloride and 1.2 ml of ketamine hydrochloride. Both drugs were mixed in the same syringe. To further control movements of the hind limbs, 20 ml solution of 2% xylocaine hydrochloride (Barrett-Hodgson, Pakistan) was injected epidurally in the sacrococcygeal space (Tanwar *et al.*, 1989).

**Fig. 2:**
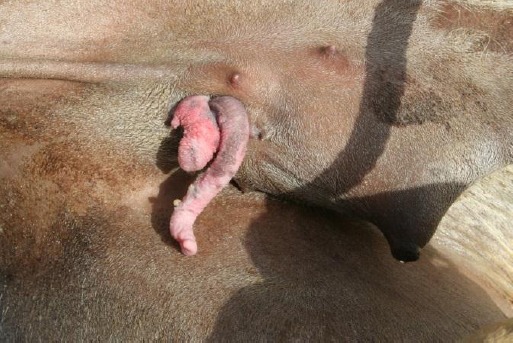
Preparation of the surgery site. Note the normal texture of the penis and the granulomatous mass at the caudal aspect of the defect.

The animal was then positioned in the left lateral recumbency. The upper hind limb was released and firmly tied to a fixed pole with a rope going around the distal third of the metatarsal region. This arrangement gave a reasonable space for surgical manipulations. The operation site was thoroughly scrubbed with Pyodine solution and clean-dried (Fig. 2).

### Corrective Surgery

**Fig. 3:**
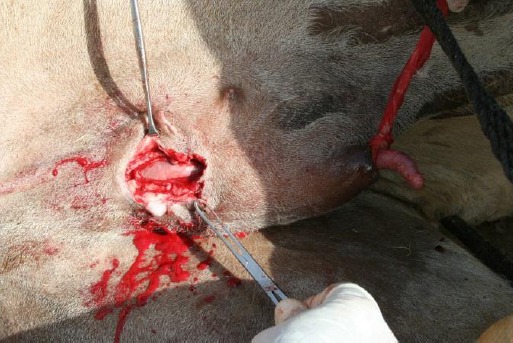
Replacement of the penis into the preputial cavity. Note the tip of the penis protruding through the preputial opening.

The granulomatous mass at the posterior edge of the defect was severed with a sharp, blunt pointed Mayo Scissors. A well-lubricated, long forceps was passed through the preputial orifice up to the blind end of the preputial cavity and the tissue was bulged over it. A guarded incision was given over the bulged tissue which was then extended to enter into the preputial cavity. The penis was released from the surrounding tissue through blunt dissection and digital manipulation and the incision was then enlarged to the extent that would allow easy replacement of the penis in the preputial cavity. A loop of a small piece of bandage was loosely applied a little above the tip of the penis. A long forceps was passed into the preputial cavity through the preputial orifice and the other end of the bandage was grasped in its jaws. The bandage was slowly pulled to replace the organ in the preputial cavity and exteriorize it through the preputial orifice (Fig. 3).

The lamina interna and the subcutaneous tissue were opposed with continuous suture lines using USP-1 Polyglycolic acid (Safil, B | BRAUN, Aesculap AG & Co. Germany) suture material with buried knots on both ends of the incision.

The skin defect was closed with USP-2 braided Supramid (Supramid, B | BRAUN, Aesculap AG & Co. Germany) suture material using horizontal mattress sutures. The suture line was protected with oxytetracycline spray (ALAMYCIN AEROSOL, Norbrook Laboratories Ltd, N. Ireland) and was cleaned and dressed as needed till removal of the skin sutures two weeks after surgery.

## Discussion

This condition was quite different from Paraphimosis; as in the latter condition, the penile tissue being quite fragile, easily undergoes edematous swelling and strangulations due to size and shape of the prepuce and constricting effect of the relatively narrow preputial orifice in camels (Choudhary *et al.*, 1981; Gahlot, 1996) as compared to that in the bovine bull (Walker, 1979).

**Fig. 4:**
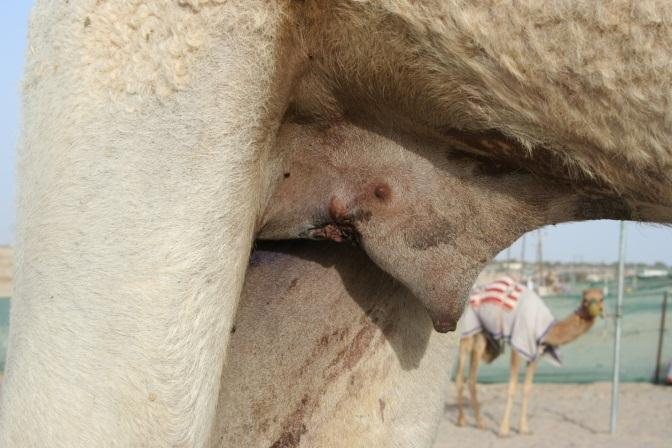
Complete retraction of the penis into the preputial cavity after the withdrawal of the effect of epidural anesthesia.

However, in the present case the penile tissue was quite normal. The animal recovered from the effect of epidural anesthesia 4 hours after surgery and stood up normally (Tanwar *et al.*, 1989). The penis that was temporarily out of the preputial orifice under the effect of epidural anesthesia just after its replacement, retracted into the cavity after the animal stood up (Fig. 4).

In cases of paraphimosis, the organ generally gets edematous and in certain cases strangulates in a relatively short period of time necessitating partial penectomy in the perineal region (Choudhary *et al.*, 1981; Gahlot, 1996). However, in this case; although the penis had been out of the preputial cavity for approximately eight months, yet it did not get strangulated and the animal never experienced any difficulty in urination.

The probable reason that these complications did not occur in this case might be that the preputial defect around the penis was not too tight to cause its strangulation. Secondly, the penis having prolapsed on one side higher up at the base of the prepuce did not had a free contact with ground even in the sitting posture that could cause injury and further complications as seen in paraphimosis (Gahlot, 1996). No postoperative complications were reported or noticed during follow up of the case for a period of two months. However, it is suggested that in such cases it is always advisable to avoid delays and the corrective surgical procedure should be undertaken at the earliest possible time to avoid any complications or irreversible damage to the organ.
